# Supplementary material for: Flexible Real-Time Polymerase Chain Reaction-Based Platforms for Detecting Deafness Mutations in Koreans: A Proposed Guideline for the Etiologic Diagnosis of Auditory Neuropathy Spectrum Disorder
Source: Diagnostics (Basel). 2020 Sep 4;10(9):672. doi: 10.3390/diagnostics10090672 (PMC7554951; doi:10.3390/diagnostics10090672)
Supplement: Supplementary file 1 [file diagnostics-10-00672-s001.zip › diagnostics-908207-SI-pdf/Supplemet Table S1.pdf]

Table S1. Mutational spectrum of prelingual auditory neuropathy spectrum disorder (ANSD) from Korean and East Asian reports [a]

U-TOP™ HL Genotyping Kit Ver2: *OTOF variants* (p.Glu841Lys, p.Arg1856Trp, p.Leu1011Pro, p.Tyr1064Ter, and p.Arg1939Gln)					
Author (Ethnicity) [Reference]	Subjects	Phenotype	Confirmed Families (biallelic *OTOF* variants)	Contribution of this kit	Others
Kim et al. (Korea) [14]	Prelingual ANSD	DFNB9	11 families (22 alleles)	p.Arg1939Gln (9/22, 40.9%)<br>p.Glu841Lys (4/22, 18.2%)<br>p.Leu1011Pro (2/22, 9.1%)<br>p.Tyr1064Ter (2/22, 9.1%)<br>p.Arg1856Trp (2/22, 9.1%)	p.Gly1845Glu (1/22, 4.5%)<br>c.4227+5G>C (1/22, 4.5%)<br>Large genomic deletion (1/22, 4.5%)
Chang et al. (Korea) [15]	Prelingual ANSD	DFNB9	5 families (10 alleles)	p.Arg1939Gln (7/10, 70.0%)<br>p.Arg1856Trp (1/10, 10.0%)	p.Glu856Lys (1/10, 10.0%)<br>Large genomic deletion (1/10, 10.0%)
Matsunaga et al. (Japan) [21]	Congenital ANSD	DFNB9	13 families (26 alleles)	p.Arg1939Gln (20/26, 76.9%)	p.Tyr474Ter (1/26, 3.8%)<br>p.Tyr1822Ter (1/26, 3.8%)<br>IVS9+5G>A (1/26, 3.8%)<br>c.1946-1965del20 (1/26, 3.8%)<br>Extension variant (1/26, 3.8%)<br>non-truncating variant (1/26, 3.8%)

Note [a]: We have summarized the genotyping profile of prelingual ANSD from East Asian papers, including ours, if at least 5 unrelated OTOF families are included
