# Supplementary figures and images for: Flexible Real-Time Polymerase Chain Reaction-Based Platforms for Detecting Deafness Mutations in Koreans: A Proposed Guideline for the Etiologic Diagnosis of Auditory Neuropathy Spectrum Disorder
Source: Diagnostics (Basel). 2020 Sep 4;10(9):672. doi: 10.3390/diagnostics10090672 (PMC7554951; doi:10.3390/diagnostics10090672)

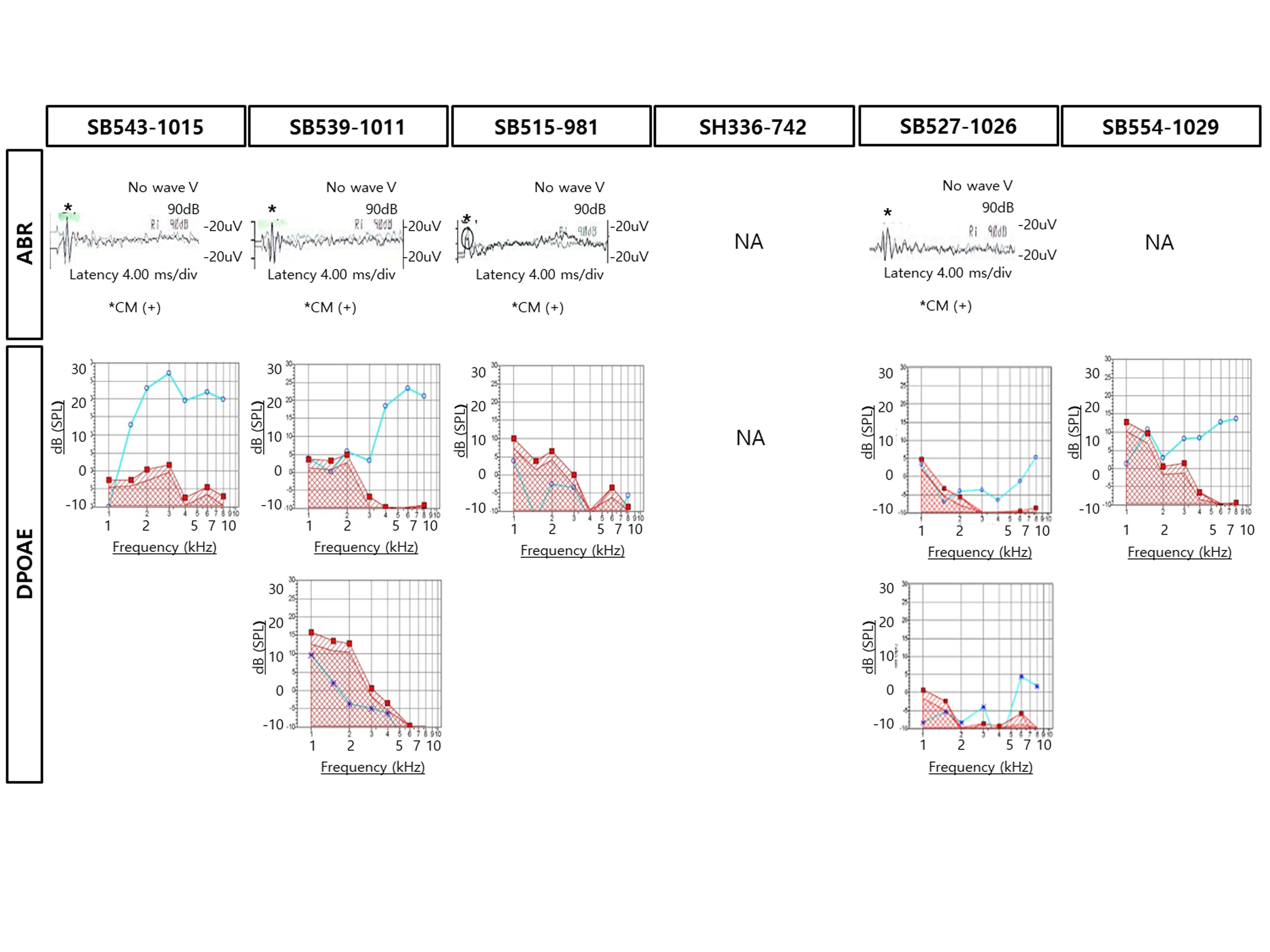

Supplement: Supplementary file 1 [file diagnostics-10-00672-s001.zip › diagnostics-908207-SI-pdf/Supplement Figure S1.tif]
